# METTL14 modulates the autophagy-pyroptosis pathway in fibroblasts by modifying BECN1 through m^6^A methylation to promote wound healing in DFUs and SYD treatment

**DOI:** 10.1007/s10565-026-10196-x

**Published:** 2026-05-01

**Authors:** Zhaowei Wang, Zongqiang Wei, Qinyao Yu, Linru Wang, Kai Liu, Hongjie Wang, Qiang Li

**Affiliations:** 1 Key Laboratory of Marine Drugs, Ministry of Education, School of Medicine and Pharmacy, Ocean University of China, Qingdao, 266003 China; 2 Department of Vascular Surgery, Qingdao Hiser Hospital Affiliated of Qingdao University (Qingdao Traditional Chinese Medicine Hospital), No.4, Renmin Road, Qingdao, 266033 Shandong Province P. R. China

**Keywords:** Diabetic foot ulcers, METTL14, Autophagy, Pyroptosis, Simiao yong'an Decoction

## Abstract

**Background:**

Diabetic foot ulcers (DFUs) are prevalent complication in diabetes. METTL14 serves as a key regulator of both autophagy and pyroptosis, both essential for the healing of DFUs. Simiao Yong'an Decoction (SYD) has demonstrated potential in promoting skin wound healing. This study investigates how METTL14 functions as a main regulator in DFU wound healing during SYD treatment and explores the underlying mechanisms.

**Methods:**

Both qPCR and western blot assays were performed to determine METTL14 and BECN1 expression. DFU rat models and fibroblasts stimulated with high glucose (HG) were used to evaluate the role of METTL14 in autophagy, pyroptosis, pro-angiogenic ability, and wound healing through both loss- and gain-of-function assays. The association between METTL14 and BECN1 was examined via MeRIP and RNA stability assays. Futhermore, the therapeutic effects of SYD on DFUs were assessed.

**Results:**

Exposure to HG reduced METTL14 levels in fibroblasts, resulting in reduced cell viability and migration, lowered autophagy, and increased pyroptosis. Increasing METTL14 expression reversed these cellular impairments and enhanced angiogenesis driven by fibroblasts. Mechanistically, METTL14 stabilized BECN1 mRNA via m^6^A modification. In a rodent DFU model, overexpression of METTL14 accelerated wound healing, improved angiogenesis, and regulated autophagy and pyroptosis; these beneficial effects were partially reversed when BECN1 was knocked down. Furthermore, treatment with SYD increased METTL14 expression, promoted wound closure, angiogenesis, and autophagy, while reducing pyroptosis; these positive outcomes were significantly reduced when METTL14 was knocked down.

**Conclusions:**

METTL14-mediated m^6^A modification of BECN1 influences autophagy, pyroptosis, and angiogenesis to enhance wound healing in DFUs. METTL14 serves as a key regulator in SYD-mediated wound repair, offering a novel therapeutic strategy for treating DFUs.

**Graphical Abstract:**

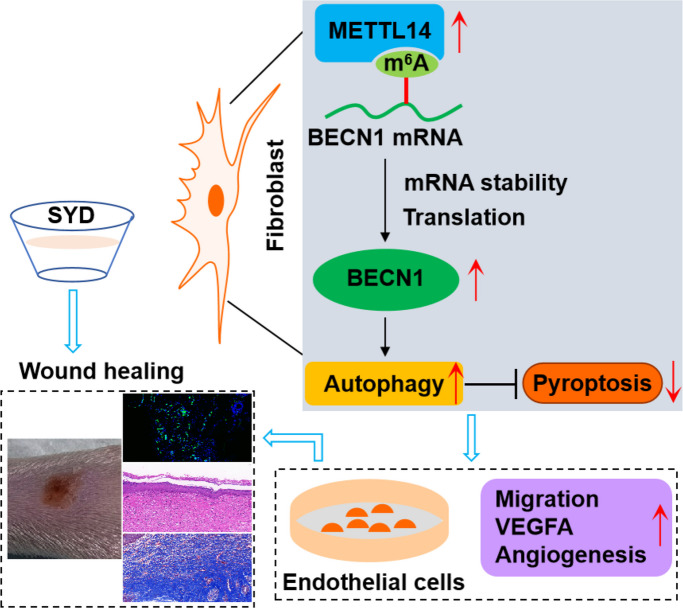

**Supplementary Information:**

The online version contains supplementary material available at 10.1007/s10565-026-10196-x.

## Introduction

Diabetic foot ulcers (DFUs) are a serious and disabling complication in diabetes, commonly related to diabetic peripheral neuropathy and foot deformities (Armstrong et al. 2017). These chronic wounds, which have an estimated global prevalence of 6.3%, account for a primary cause of diabetes related hospitalization (Hicks and Selvin 2019). Beyond the immediate clinical consequences, DFUs place a significant burden on patients by causing extended hospital stays, increasing healthcare expenses, and reducing life quality (Armstrong et al. 2017; Zhang et al. 2017). The suboptimal efficacy of current therapeutic approaches highlights the critical requirement to discover novel therapeutic targets and refining clinical interventions to improve DFU healing.

Fibroblasts represent the primary cellular population of the dermis and are integral to the regulation of every stage of the wound repair process. Fibroblasts are responsible for the synthesize and release key repair factors such as VEGF, and participate in modulating of inflammatory response (Li et al. 2024). In DFUs, chronic hyperglycemia induces substantial dysfunction in fibroblast activity, characterized by impaired proliferation and migration. These alterations contribute to inadequate wound angiogenesis, thereby impeding effective wound repair (Richard et al. 2011; Voza et al. 2024). Notably, evidence from co-culture models indicates that fibroblasts significantly enhanced the motility and capillary-like structure formation of endothelial cells, which highlight fibroblasts act as essential mediators of angiogenesis during wound healing (Chen et al. 2025; Huang et al. 2020). Therefore, therapeutic strategies aimed at restoring or targeting dysfunctional fibroblasts hold significant potential for improving healing outcomes in DFUs.

Recent developments in the field of epitranscriptomics have underscored the pivotal role of N6-methyladenosine (m^6^A) RNA methylation as a critical mechanism governing gene expression and diverse cellular functions (Wang et al. 2014; Yang et al. 2018). Investigations have demonstrated a reduction of the m^6^A reader protein YTHDC1 in the epidermis of diabetic murine models, which adversely impacts autophagy progression (Liang et al. 2022). The m^6^A methyltransferase METTL3 has been reported to facilitate angiogenic processes in DFUs by modulating the m^6^A modification of VEGFC (Zhou et al. 2021). Additionally, methyltransferase-like 14 (METTL14), an another m^6^A writer enzyme, has been associated with multiple diabetic complications, including nephropathy and cardiomyopathy (Geng et al. 2022; Li et al. 2021; Meng et al. 2022). Emerging evidence indicate that METTL14 may regulate fibroblast activity by modulating gene expression programs related to extracellular matrix remodeling, inflammation, and angiogenesis (Geng et al. 2022). However, the mechanistic function of METTL14 in the development of DFUs, particularly regarding fibroblast function, remains insufficiently characterized.

Cellular dysfunction in DFUs is partly driven by the interplay between autophagy and pyroptosis. Autophagy participates in multiple stages of wound repair, and its dysregulation has been implicated in the delayed healing characteristic of diabetes (Ren et al. 2022). Activation of autophagy has been demonstrated to suppress senescence in dermal fibroblasts and to enhance wound healing in DFU rat models (Luo et al. 2025). Pyroptosis, an inflammasome activated programmed cell death mediated, contributes to the persistent inflammatory milieu that hinders healing in DFUs (Mu et al. 2022). Inhibition of fibroblast pyroptosis has been reported to enhance cutaneous wound repair in diabetic rats (Zhao et al. 2025). The concept of the "autophagy-pyroptosis axis" encapsulates the intricate crosstalk between these pathways and is increasingly recognized as a critical regulatory nexus governing fibroblast function (Lee et al. 2020). Autophagy within fibroblasts may inhibit pyroptosis, augment the secretion of angiogenic factors, and thereby facilitate endothelial cell angiogenesis (Ren et al. 2022). Additionally, METTL14 has been proved as a modulator that activates autophagy (He et al. 2022) and suppresses pyroptosis (Meng et al. 2022). Nonetheless, the mechanisms by which METTL14 regulates this axis in DFUs, and thereby influences fibroblast-mediated angiogenesis and wound healing, remain incompletely understood.

Simiao Yong'an Decoction (SYD) is a common traditional Chinese medicine (TCM) formulation (Liao et al. 2022). Employing advanced analytical methods such as UPLC-Q-TOF–MS/MS, researchers have identified multiple bioactive constituents in SYD, including chlorogenic acid, forsythoside A, liquiritin, calycosin-7-glucoside, and ginsenoside Rg1. These compounds are integral to quality control processes and are associated with the formulation's therapeutic properties (Sun et al. 2022; Wang et al. 2023). Accumulating studies denmostrate that SYD modulates various physiological processes, including inflammation, angiogenesis, oxidative stress, lipid metabolism, and blood rheology (Chen et al. 2021; Wang et al. 2015; Zhu et al. 2020). Clinical trials within the TCM framework have demonstrated SYD's efficacy in treating peripheral vascular diseases, diabetes, and coronary heart disease (Du et al. 2021; Liao et al. 2022; Wang et al. 2015; Zhu et al. 2020). Experimental researches have further shown that SYD administration enhances the sensitivity of insulin, reduces blood glucose levels, stabilizes atherosclerotic plaques, and consequently improves cardiac function in diabetic murine models (Li et al. 2020; Peng et al. 2012). Recent study suggests that SYD may facilitate wound healing in DFUs via the Wnt signaling (Zhao et al. 2017). However, a comprehensive mechanistic elucidation of SYD's therapeutic effects in DFUs remains lacking, particularly regarding its potential interactions with METTL14 and the autophagy-pyroptosis axis within fibroblasts.

The work seeks to uncover the molecular mechanisms of METTL14 in modulating fibroblast activity in the context of DFUs. Our findings indicated that METTL14 serves as a pivotal regulator of the autophagy-pyroptosis axis via enhancing the stability of BECN1 mRNA via m^6^A methylation, consequently enhancing fibroblast-mediated angiogenesis and facilitating wound repair. Additionally, SYD contributes to the healing of DFUs through the regulation of the METTL14-BECN1 signaling pathway.

## Materials and methods

### Animal model and experimental procedures

Sprague Dawley rats (female, 150–200 g, 4 to 6 weeks) were obtained from Shanghai SLAC (China). Ethical approval for all experimental protocols was obtained from the Ethics Committee of our Hospital (2024HC10LS001). Establishment of rat diabetes models were performed by initially subjected to a high-fat, high-sucrose diet over a four-week period to induce insulin resistance. Subsequently, streptozotocin (STZ) was administrated intraperitoneally as a single injection at a concentration of 0.45% (35 mg/kg; Sigma-Aldrich). Seventy-two hours post-injection, rats with fasting blood glucose (FBG) levels above 16.7 mM were considered as diabetic. After confirming the induction of diabetes, 5 mm diameter full-thickness skin wounds in rats were made from the dorsal aspect of the hind feet.

To inhibit METTL14 or BECN1 expression, short hairpin RNAs (shRNAs) targeting METTL14 or BECN1 were designed and packaged into Adeno-Associated Virus serotype 2/9 vectors (AAV-shMETTL14 or AAV-shBECN1; GenePharma, Shanghai, China). For METTL14 overexpression, METTL14 complementary DNA (cDNA) was cloned into the pcDNA3.1 vector and subsequently packaged into AAV particles (AAV-oe-METTL14). All AAV vectors were produced and purified by GenePharma following their established protocols, and the viral titers were standardized at 1 × 10^12 viral genomes (vg)/mL. The respective AAV vector solution (50 µL) was administered via intradermal injection at four equidistant sites surrounding the wound margin (12.5 μL per site) using a 30-gauge needle. These injections were performed two days post-induction of DFUs.

For SYD treatment, DFU rats received the treatment with SYD at a dosage of 10 mL/kg administered via gastric gavage twice daily for 14 days (Zhao et al. 2017). The DFU rats were randomly assigned to the control group, which was received an equivalent volume of normal saline. No signs of toxicity (e.g., weight loss, behavioral abnormalities) were observed during treatment, further supporting the safety of this dosage. The SYD formulation was prepared by decocting 90 g of honeysuckle, 90 g of Genuine Ginseng, 60 g of angelica, and 30 g of licorice in water at Qingdao Hiser Hospital Affiliated of Qingdao University. The resulting decoction was then concentrated to a final density of 5.6 g/mL. To ensure consistency and reproducibility, the final decoction was subjected to chemical profiling using UPLC-MS/MS analysis. This analysis identified a total of 1653 components in SYD, including Verbascoside, 3-Feruloylquinic acid, Isoliquiritin apioside, Isorhamnetin-3-O-glucoside, Harpagoside, Vicenin 2, Abscisic acid, 3,4-Dicaffeoylquinic acid, and N-Acetyl-L-tryptophan. The total ion chromatogram is shown in Supplementary Fig. 1.

The images of wounds were obtained on days 0, 7, and 14, and healing rates were quantified with ImageJ. On the 14th day after wound induction, skin tissues adjacent to the wound sites were collected for subsequent biochemical analyses.

### Histological analysis

Skin tissues embedded in paraffin were sectioned into 5 μm slices, which were subsequently analyzed by hematoxylin and eosin (HE), Masson's trichrome, and immunofluorescence staining. Immunofluorescence staining was conducted using CD31 (bs-20322R, Bioss Antibodies, Beijing, China), LC3B (bsm-60842R, Bioss Antibodies), NLRP3 (bs-10021R, Bioss Antibodies), and Vimentin (bs-8533R, Bioss Antibodies) antibodies. Histological observations were performed using a Nikon 4500 camera (Tokyo, Japan). Quantitative analyses of collagen fiber deposition and vascular density were performed utilizing ImageJ software. For each sample, three non-overlapping sections were analyzed, and five random fields per section were selected for quantification at 4 × or 10 × magnification. Eight mice per group were included in the analysis. Image acquisition and quantitative analyses were conducted under blinded conditions to reduce potential bias.

### Cell culture and experiment design

Rat dermal fibroblasts (CP-R086) and endothelial cells (CP-R196) were obtained from Procell (Wuhan, China) and maintained under standard culture conditions. Cell transfection for overexpression of METTL14 or inhibition of BECN1 were conducted using Lipofectamine™ LTX (15338100, Invitrogen). To simulate a diabetic mellitus cellular model, fibroblasts (70–80% confluence) were exposed to high glucose conditions (HG; 25 mM) for 48 h in serum-free medium. Subsequently, the culture medium of fibroblasts was collected and centrifuged at 300 × g for 10 min at 4 ℃ to eliminate cells and debris. The supernatant was further centrifuged at 2,000 × g for 15 min to eliminate fine cell fragments. The resulting conditioned medium, obtained by filteration through a 0.22 μm membrane filter, was stored at −20 ℃ or −80 ℃ until further use. To assess the impact of METTL14 on cell migration and tube formation relevant to DFUs, endothelial cells were incubated with/without conditioned medium derived from the treated fibroblasts for 24 h.

### qPCR

Total RNA from skin samples or cultured cells was isolated using Trizol reagent (Invitrogen), followed by reverse transcription into cDNA with the PrimeScript™ 1 st Strand cDNA Synthesis Kit (Takara). qPCR was subsequently carried out using the SYBR Green PCR Master Mix (Takara). Relative gene expression levels were determined by the ∆∆CT method and normalized to the GAPDH mRNA expression. The primer sequences used in this study are listed below. METTL14 (forward: TTT CTC TGG TGT GGT TCT GG, reverse: AAG TCT TAG TCT TCC CAG GAT TG), BECN1 (forward: GTG CTC CTG TGG AAT GGA AT, reverse: TGC ACA CAG TCC AGA AAA GC), GAPDH (forward: ACC AGG TAT CTG CTG GTT G, reverse: TAA CCA TGA TGT CAG CGT GGT).

### Western blotting

Total protein from cells or skin tissues was isolated using RIPA lysis buffer. Following electrophoresis, protein samples were transferred onto polyvinylidene difluoride (PVDF) membranes (Millipore, Jaffrey, NH, USA). After blocking with 5% skimmed milk for one hour, the membranes were incubated with primary antibodies at 4 ℃ overnight. The antibodies employed in this study included METTL14 (PA5-117138, 1:1000, Invitrogen), VEGFA (ab214424, 1:1000, Abcam), cleaved Caspase-1 (AF4022, 1:500, Affinity, Jiangsu, China), ASC (ab307560, 1:1000, Abcam), NLRP3 (ab263899, 1:1000, Abcam), p62 (#39749, 1:1000, Cell Signaling Technology, Danvers, MA, USA), GSDMD-N (DF13758, 1:1000, Affinity), BECN1 (#3738, 1:1000, Cell Signaling Technology), LC3B (#43566, 1:1000, Cell Signaling Technology), and GAPDH (PA1-16777, 1:5000, Invitrogen). Visualization of protein signals was achieved through ECL detection (Bio-Rad Laboratories), followed by densitometric quantification with ImageJ software.

### Quantification of m^6^A level

Fibroblasts were seeded into 96-well plates for subsequent analyses. Total RNA was extracted with Trizol reagent. Subsequently, the integrity and quality of the isolated RNA were assessed. The level of m^6^A modification in the fibroblast RNA samples was then quantified by utilizing the m^6^A Quantification Kit (ab185912, Abcam).

### Methylated RNA immunoprecipitation (MeRIP) assay

Fibroblasts were seeded in 6-well plates and subsequently transfected with either oe-METTL14 or oe-NC. Total mRNA was subsequently purified using an mRNA Isolation Kit (Eide Technology, Beijing, China). For immunoprecipitation, rotein A/G beads were pre-incubated for one hour with anti-m^6^A antibody (ab151230, 1:500, Abcam) or IgG control antibody (ab182931, 1:100, Abcam) in IP buffer. The bead-antibody complexes were subsequently mixed with the purified mRNA and rotated at 4 ℃ overnight. After wash and elution,, the precipitated RNA was collected, and BECN1 mRNA were quantified by qPCR.

### mRNA stability assay

Rat fibroblasts were seeded in 6-well plates and were transfected with a plasmid designed to overexpress METTL14 and incubated for 48 h. Subsequently, T T transcription was arrested using Actinomycin D (5 μg/mL), followed by cell collection at the indicated time points. Total RNA was isolated from the cells at 0, 3, and 6 h post-treatment using Trizol reagent. The mRNA expression of BECN1 were detected by qPCR.

### Preparation of SYD-containing serum for in vitro experiments

Rats were randomly assigned to two groups: the control serum group and the SYD-treated serum group (*n* = 10 per group). The SYD group received SYD (20 g·kg⁻^1^·day⁻^1^) by oral gavage (calculated based on crude drug weight) twice daily for one week, while the control group received an equal volume of normal saline. Blood collection was performed 1 h after the final administration under anesthesia via the abdominal aorta. Serum was obtained by centrifugation at 3000 rpm for 10 min, followed by heat inactivation at 56 °C for 30 min. The serum was then filtered through a 0.22 µm sterile membrane. A 10% concentration of SYD-containing serum was utilized in the culture medium of HG-treated fibroblasts for assessing its effects on autophagy and pyroptosis-related pathway.

### VEGFA level detection

VEGFA levels in conditioned medium derived from fibroblasts were detected using a Rat VEGFA ELISA Kit (E-EL-R2603, Elabscience, Wuhan, China).

### CCK-8

CCK-8 method was conducted to determine the viability of the cells following treatment. Rat dermal fibroblasts were seeded and transfected with either a METTL14 overexpression plasmid or an oe-NC control plasmid, followed by exposure to HG conditions for 48 h. Subsequently, CCK-8 reagent (10 μL per well, ab228554, Abcam) was applied, and cells were incubated for 2 h at 37 ℃. Absorbance at 450 nm was subsequently quantified using a microplate reader.

### Tube formation assay

The Angiogenesis Assay Kit (ab204726, Abcam) was utilized to evaluate the capacity of endothelial cells to generate capillary-like networks. In brief, an extracellular matrix solution was applied to a sterile culture plate and incubated at 37 ℃ for one hour to allow gel polymerization. Thereafter, endothelial cells (2 × 10^4^ cells per well) were seeded onto the polymerized gel and incubated for 12 h to facilitate tube formation. The cells were then washed and stained for 30 min. Subsequently, images were obtained using an Olympus microscope, and the number of mesh-like structures was quantitatively analyzed.

### Wound healing assay

The migratory ability of fibroblasts and endothelial cells was determined using a wound healing test. Specifically, fibroblasts or endothelial cells were seeded into 12-well plates and subjected to treatments according to the experimental protocol. After culturing to approximately 100% confluence, a linear scratch was created crossthe cell monolayer to simulate an artificial wound. Cell migration into the resulting gap was captured at 0 and 24 h using a light microscope equipped with imaging software.

### Statistical analysis

Data are expressed as the mean ± standard deviation. The statistical analyses were performed using GraphPad Prism software (version 10.2.3). Differences were analyzed using Student's t-test or one-way analysis of variance (ANOVA) with Tukey's post-hoc test. In vitro experiments were repeated independently at least three times. In the animal study, each group consisted of eight rats. While a formal power calculation was not performed, the cohort size was guided by relevant published literature and prior laboratory experience. The investigator was blinded to group assignments during both the experimental procedures and outcome assessments.

## Results

### Regulation of autophagy and pyroptosis by METTL14 in HG-treated fibroblasts

**Fig. 1:**
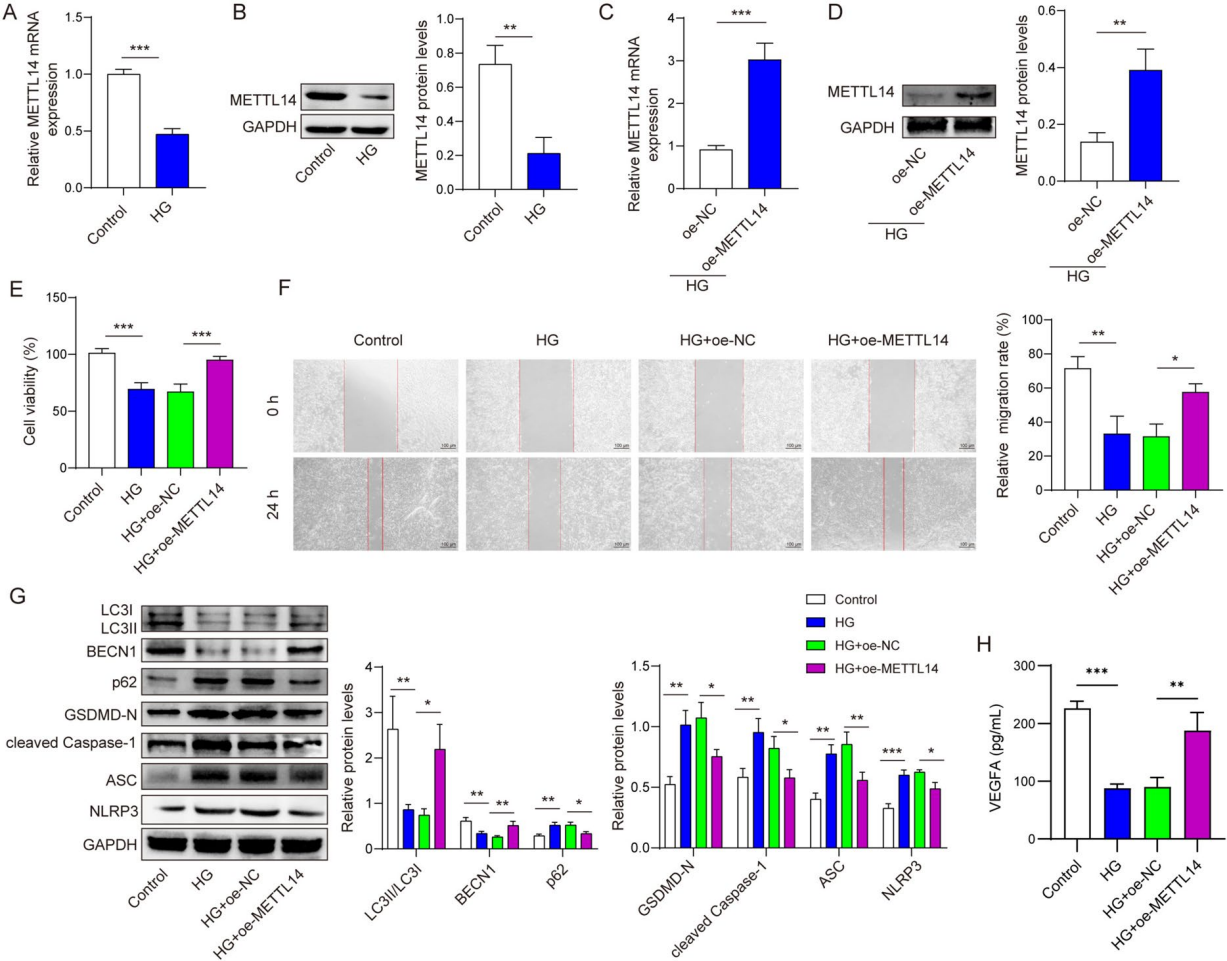
Regulation of autophagy and pyroptosis by METTL14 in fibroblasts exposed to HG conditions. Fibroblasts were treated with HG (25 mmol/L) for 48 h. **A** qPCR analysis was performed to determine METTL14 gene expression in fibroblasts. **B** Western blot analysis was conducted to evaluate METTL14 protein levels in fibroblasts subjected to HG treatment. Fibroblasts were transfected with a METTL14 overexpression plasmid (oe-METTL14) and subsequently exposed to HG (25 mM) or control conditions for 48 h. **C** METTL14 gene expression was quantified by qPCR following transfection. **D** Western blotting was used to assess METTL14 protein expression post-transfection. **E** Cell viability was assessed using the CCK-8 assay. **F** Fibroblast migratory capacity was evaluated via wound healing assay. Scale bar: 100 µM, magnification: 4 ×. **G** Western blot analysis was performed to measure the expression levels of autophagy-related proteins LC3 I/II, BECN1, p62, and pyroptosis-associated proteins GSDMD-N, NLRP3, cleaved Caspase-1, and ASC in fibroblasts. (**H**) The levels of VEGF in the conditioned medium were determinate by ELISA. *n* = 3. *p < 0.05, **p < 0.01, ***p < 0.001

In this study, we found that HG stimulation led to a pronounced decrease in METTL14 expression in fibroblasts, as evidenced by both transcriptional and translational analysis (Fig. 1A, B). To elucidate the effect of METTL14 on modulating autophagy and pyroptosis under HG conditions, fibroblasts were transfected with a METTL14 overexpression plasmid (oe-METTL14). This intervention resulted in a marked upregulation of METTL14 gene and protein expression in HG-treated fibroblasts (Fig. 1C, D). Functionally, HG exposure was found to impair cell viability and migration; however, METTL14 overexpression significantly enhanced these cellular functions (Fig. 1E, F). At the molecular level, HG treatment led to a reduction in the LC3 II/I ratio and BECN1 expression, along with an increase in p62 levels, indicative of suppressed autophagy (Fig. 1G). Concurrently, HG stimulation induced the increases of GSDMD-N, NLRP3, cleaved Caspase-1, and ASC (Fig. 1G). Notably, METTL14 overexpression effectively counteracted these HG-induced alterations in autophagy and pyroptosis markers (Fig. 1G). Moreover, HG significantly decreased the secretion of VEGFA compared to control group, METTL14 overexpression restoring VEGFA level observed under normal glucose conditions (Fig. 1H). Collectively, these findings suggest that METTL14 facilitates autophagic processes while inhibiting pyroptotic pathways in fibroblasts exposed to HG conditions.

### METTL14 facilitates fibroblast-mediated endothelial cell angiogenesis

**Fig. 2:**
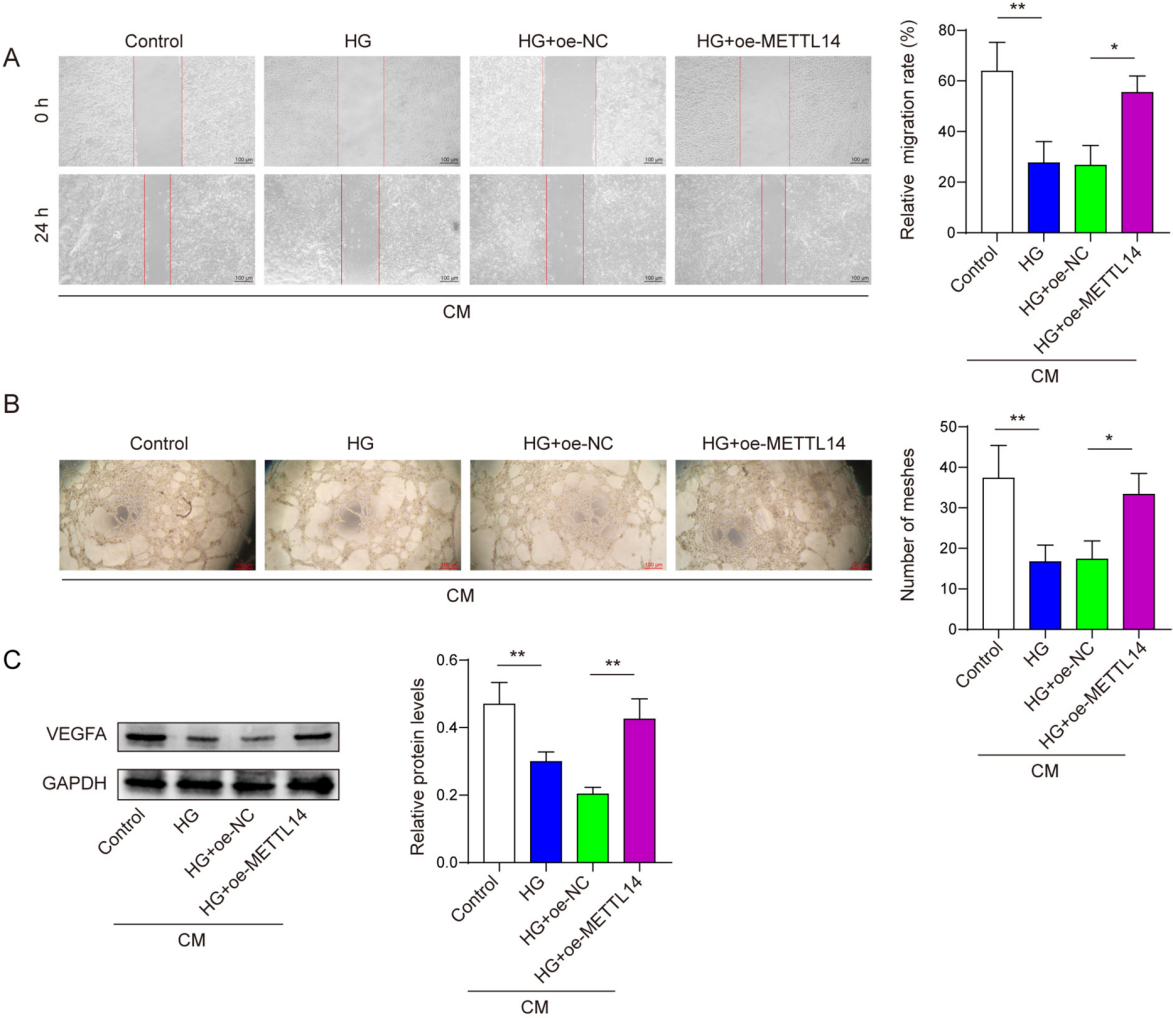
The impact of METTL14 on fibroblast-mediated angiogenesis in vitro. Endothelial cells were incubated with conditioned medium (CM) derived from fibroblasts transfected with an METTL14 overexpression plasmid (oe-METTL14) and subsequently exposed to HG (25 mM) or control conditions for 48 h. **A** Endothelial cell migration was evaluated using a wound healing assay. Scale bar: 100 µM, magnification: 4 ×. **B** Angiogenic capacity of endothelial cells was assessed through a tube formation assay. Scale bar: 100 µM, magnification: 4 ×. **C** VEGFA protein expression levels in endothelial cells were determined by western blot analysis. *n* = 3. *p < 0.05, **p < 0.01

The role of METTL14 in fibroblast-driven endothelial cell migration and tube formation was evaluated by treating endothelial cells with conditioned media from fibroblasts transfected with oe-METTL14 or oe-NC under both HG and normoglycemic conditions. Endothelial cell migratory capacity was markedly diminished when cultured with conditioned medium from HG-treated oe-NC fibroblasts. In contrast, conditioned medium from HG-treated oe-METTL14 fibroblasts significantly mitigated this reduction in migration (Fig. 2A). Similarly, tube formation by endothelial cells was impaired following exposure to conditioned medium from HG-treated oe-NC fibroblasts; however, conditioned medium from oe-METTL14 fibroblasts under HG conditions enhanced tube formation relative to the oe-NC group (Fig. 2B). Moreover, VEGFA expression in endothelial cells was downregulated upon treatment with conditioned medium from HG-treated oe-NC fibroblasts, whereas this decrease was partially reversed by conditioned medium from oe-METTL14 fibroblasts (Fig. 2C). Therefore, these results indicate that METTL14 augments fibroblast-mediated angiogenic processes in endothelial cells.

### METTL14 enhances BECN1 mRNA stability via m^6^A modification

**Fig. 3:**
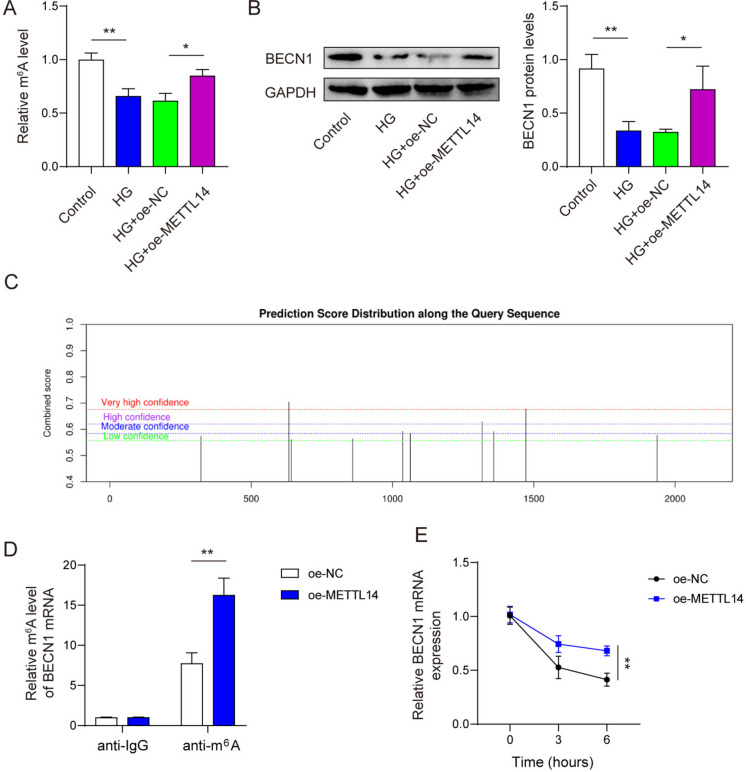
METTL14 promotes the stability of BECN1 mRNA via m^6^A modification. Fibroblasts were transfected with a METTL14 overexpression plasmid (oe-METTL14) and subsequently treated with or without HG (25 mM) for 48 h. **A** The global m^6^A modification levels in fibroblasts were quantified using an m^6^A RNA Methylation Quantification Kit. **B** BECN1 protein expression in fibroblasts was evaluated by western blot analysis. **C** The SRAMP tool (http://www.cuilab.cn/sramp/) was employed to predict potential m^6^A modification sites on BECN1 mRNA. **D** The influence of METTL14 on m.^6^A modification of BECN1 mRNA was confirmed through MeRIP assays. **E** The impact of METTL14 on the stability of BECN1 mRNA was assessed by qPCR. *n* = 3. *p < 0.05, **p < 0.01

Given the established role of METTL14 in regulating BECN1 expression and autophagy in fibroblasts (Fig. 1G), we sought to determine whether METTL14 modulates BECN1 expression through m^6^A RNA methylation, considering BECN1's critical function in autophagosome formation. Analysis revealed a remarkable reduction in m^6^A levels in fibroblasts treated with HG compared to controls (Fig. 3A). Conversely, METTL14 overexpression markedly elevated m^6^A modification levels in HG-treated fibroblasts (Fig. 3A). Correspondingly, BECN1 expression was diminished in HG-treated fibroblasts relative to controls, an effect that was partially reversed following METTL14 overexpression (Fig. 3B). A search of the SRAMP database identified high-confidence m^6^A modification sites within the BECN1 transcript (Fig. 3C). MeRIP assays further substantiated that METTL14 overexpression augmented m^6^A modification of BECN1 mRNA (Fig. 3D). Furthermore, METTL14 overexpression significantly enhanced BECN1 mRNA stability (Fig. 3E). To further determine whether METTL14 regulates BECN1 via the m^6^A-dependent manner, a predicted m^6^A modification site in BECN1 mRNA (position 633, AGACC) was mutated to AGGCC. Functional analysis showed that wild-type BECN1 overexpression effectively restored autophagy and suppressed pyroptosis in METTL14-deficient fibroblasts. In contrast, the mutant BECN1 exhibited a significantly reduced ability to rescue these effects, as evidenced by decreased LC3 II/I ratio, increased p62 accumulation, and enhanced levels of pyroptosis-associated proteins (Supplementary Fig. 6). Collectively, these results indicate that METTL14 regulates BECN1 expression through its m^6^A methyltransferase activity, thereby influencing mRNA stability.

### METTL14 facilitates wound healing via BECN1 in DFU rat models

**Fig. 4:**
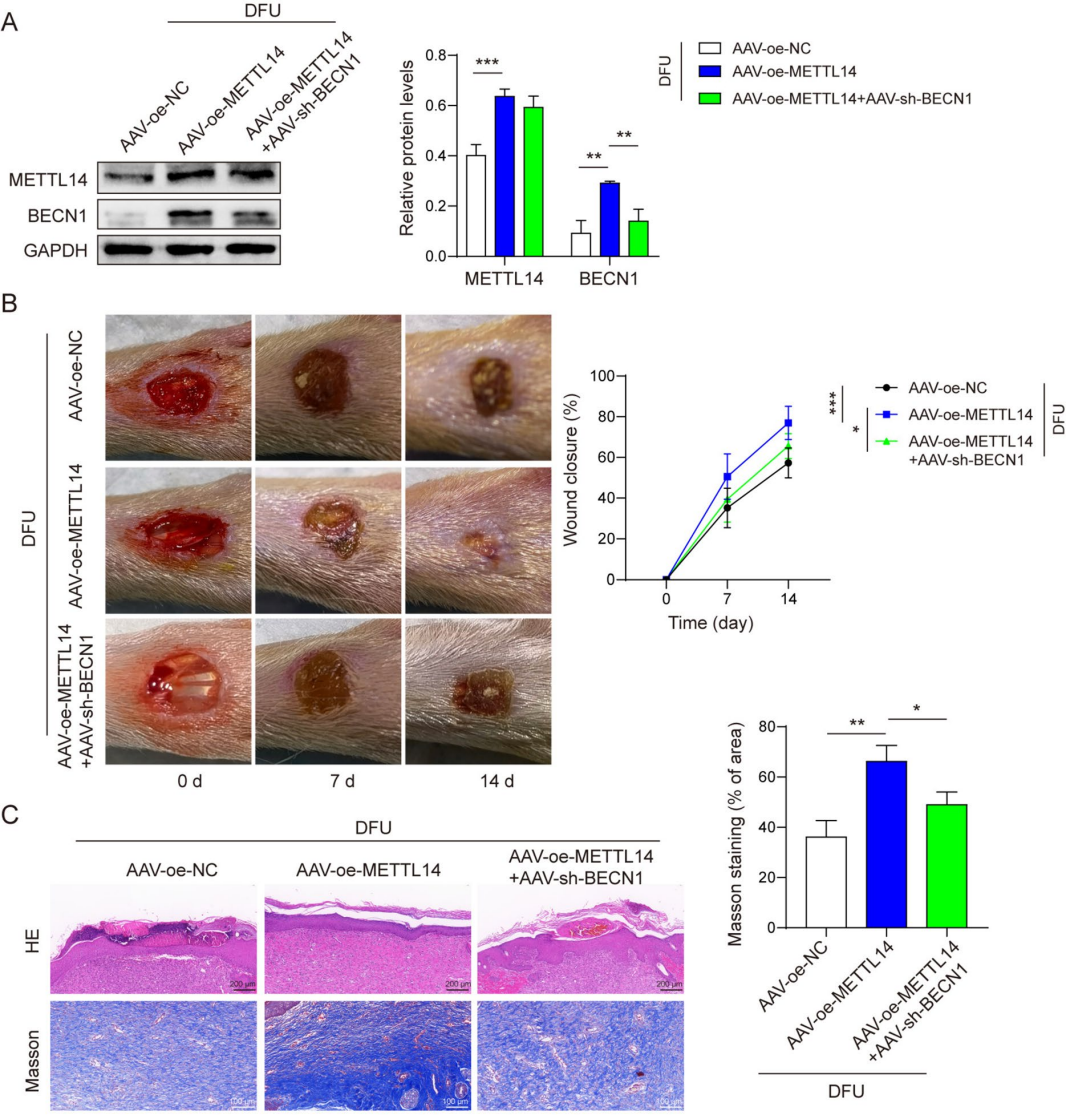
METTL14 facilitates wound healing in DFU rats through BECN1. A rat model of DFU was established and treated with either an AAV mediated overexpression METTL14 plasmid (oe-METTL14) or AAV mediated BECN1 knockdown shRNA (sh-BECN1). **A** Western blot analysis was performed to assess the protein expression levels of METTL14 and BECN1 in the skin tissues of DFU rats. **B** The wound areas of DFU rats were monitored and documented. **C** HE (Scale bar: 200 µM, magnification: 4 ×) and Masson (Scale bar: 100 µM, magnification: 10 ×) staining, were conducted on skin tissue samples from the wound sites to assess pathological changes and collagen fiber deposition. Each experimental group consisted of eight rats (*n* = 8). *p < 0.05, **p < 0.01, ***p < 0.001

To elucidate the effect of METTL14 on accelerating wound repair via BECN1 in DFU rats, an AAV vector was employed to induce overexpression of METTL14, while BECN1 expression was suppressed through the administration of AAV-mediated shRNA specifically targeting BECN1. We firstly confirmed that the expression METTL14 in the wound skin tissues of DFU rats was significantly decreased than the normal mice (Supplementary Fig.  2A). Immunofluorescence co-staining revealed that METTL14 expression was predominantly colocalized with Vimentin-positive fibroblasts in wound tissues (Supplementary Fig. 5), suggesting that METTL14 modulation mainly occurs in fibroblasts in DFU rat model. METTL14 overexpression led to a significant upregulation of BECN1 in the skin tissues of DFU rats; conversely, BECN1 knockdown partially attenuated this effect (Fig. 4A). Functionally, compared to the non-treated DFU rats, METTL14 overexpression markedly decreased wound area and enhanced wound closure in DFU rats (Supplementary Fig. 2B and Fig. 4B), even partially restoring the healing rate toward that observed in normal rats (Supplementary Fig. 2B). Notably, the suppression of BECN1 partially abrogated the beneficial effects conferred by METTL14 overexpression (Fig. 4B). Histological analysis revealed that METTL14 overexpression promoted wound repair, reduced inflammatory cell infiltration and enhanced neovascularization, while data from Masson staining showed an increased collagen deposition in DFU rats. However, these histological improvements were partially reversed upon BECN1 silencing (Fig. 4C). Together, these findings suggest that METTL14 facilitates wound healing in DFU rats through a mechanism involving BECN1.

### METTL14 accelerates angiogenesis by modulating the BECN1-autophagy-pyroptosis axis in DFU rat models

**Fig. 5:**
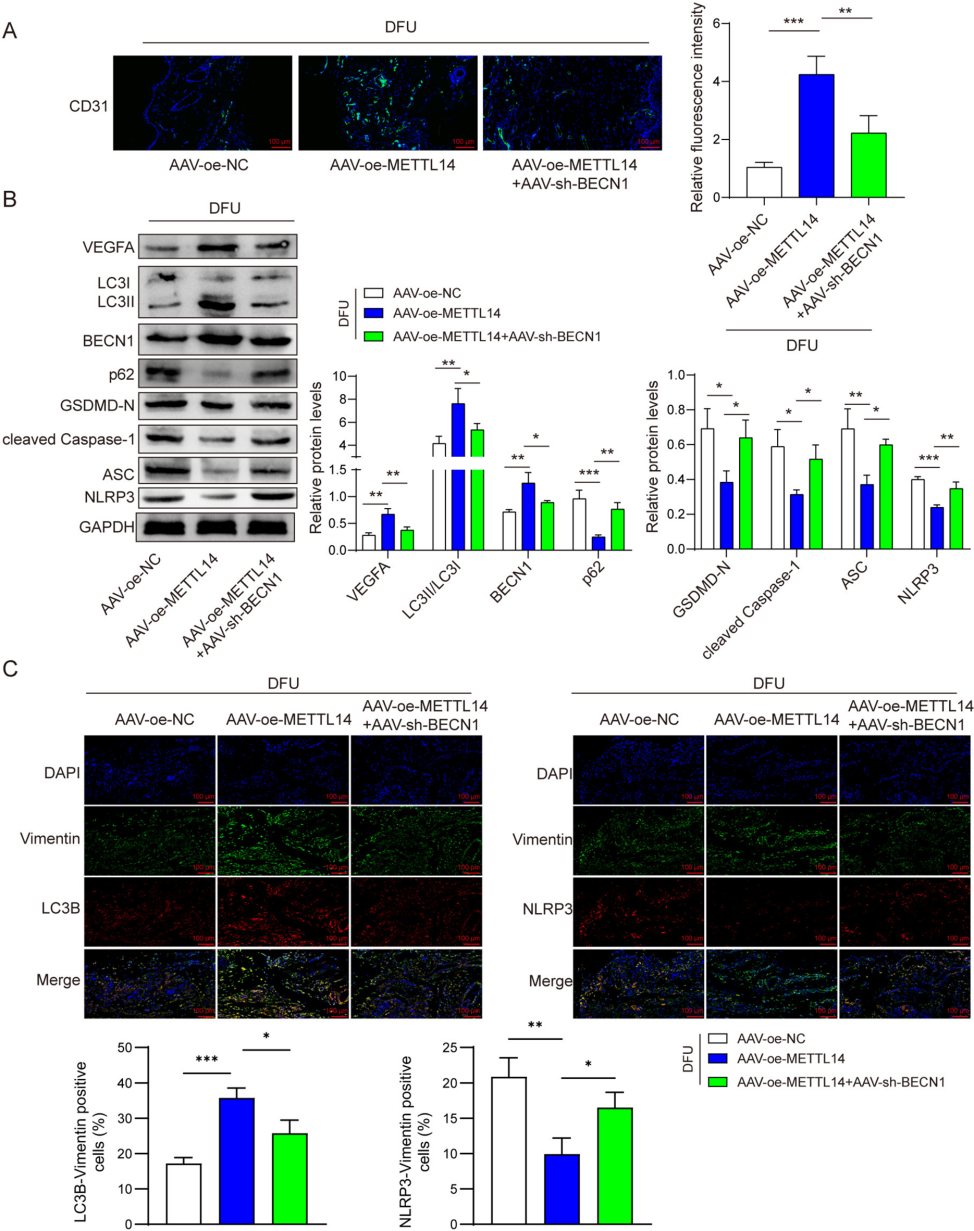
The regulatory role of METTL14 in autophagy, pyroptosis, and angiogenesis through its interaction with BECN1 in a DFU rat model. The DFU model was established in rats, which were treated with either an AAV mediated overexpression METTL14 plasmid (oe-METTL14) or AAV mediated BECN1 knockdown shRNA (sh-BECN1). **A** Immunofluorometric analysis of CD31 in skin tissue samples. Scale bar: 100 µM, magnification: 10 ×. **B** The protein expression levels of VEGFA, LC3 I/II, BECN1, p62, GSDMD-N, NLRP3, cleaved Caspase-1, and ASC in the skin tissues of DFU rats were assessed by western blotting. **C** Representative immunofluorescence images showing LC3B and NLRP3 expression in in fibroblasts of wound tissues. Nuclei were stained with DAPI (blue). Co-localization with Vimentin-positive cells indicates that these changes occur in fibroblasts. Scale bar: 100 μm. Magnification: 10 ×. Each experimental group consisted of eight rats (*n* = 8). *p < 0.05, **p < 0.01, ***p < 0.001

To investigate the role of METTL14 in promoting wound repair in DFUs via the regulation of autophagy, pyroptosis, and angiogenesis via BECN1, experiments were conducted using AAV engineered for METTL14 overexpression alongside BECN1-specific shRNA AAV in DFU rat models. Overexpression of METTL14 significantly enhanced CD31 expression relative to control groups, indicating an increase in angiogenesis within the DFU rats (Fig. 5A). Conversely, suppression of BECN1 partially attenuated the angiogenic effects induced by METTL14 overexpression (Fig. 5A). At the molecular level, inhibition of BECN1 reversed the METTL14-induced upregulation of VEGFA, LC3 II/I, and BECN1 in skin tissues, while also counteracting the METTL14-mediated downregulation of p62 in the same tissues (Fig. 5B). Furthermore, knockdown of BECN1 mitigated the reductions in pyroptosis-related markers, including GSDMD-N, cleaved Caspase-1, NLRP3, and ASC, that were otherwise observed following METTL14 overexpression in DFU rat skin tissues (Fig. 5B). Immunofluorescence staining showed METTL14 overexpression increased LC3B level and decreased NLRP3 expression in fibroblasts, while knockdown of BECN1 reverse these changes observed in METTL14 overexpression DFU rats (Fig. 5C). Collectively, these findings demonstrate that METTL14 modulates autophagy, pyroptosis, and angiogenesis through targeting BECN1 in the context of DFUs in rat models.

### SYD modulates autophagy, pyroptosis, and angiogenesis in DFU rat models

**Fig. 6:**
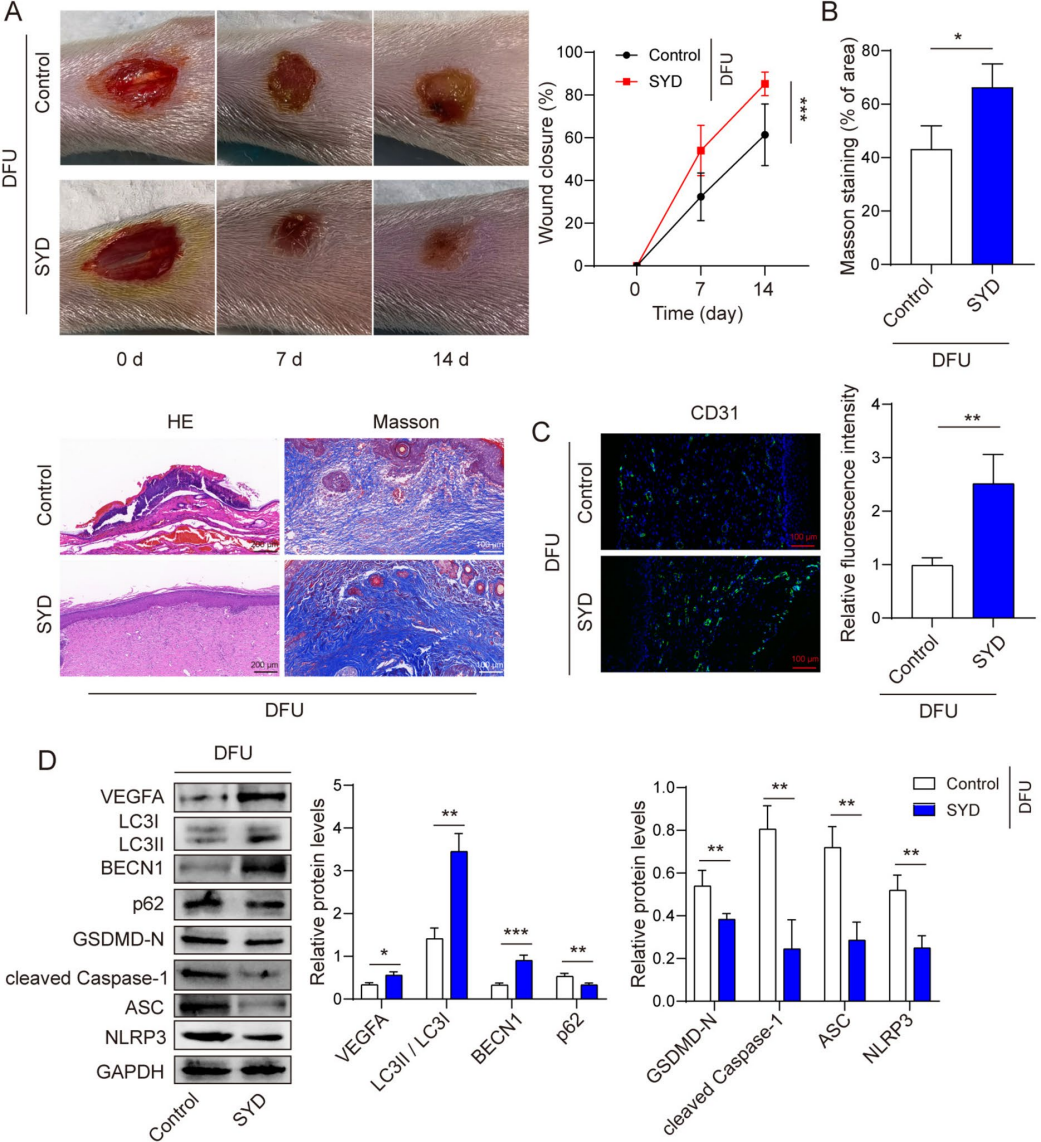
The impact of SYD on autophagy, pyroptosis and angiogenesis in DFU rat models. DFU rats received SYD treatment (10 mL/kg) via gastric gavage. **A** Wound area progression in DFU rats was documented. **B** Histological analyses of skin tissues were performed using HE (Scale bar: 200 µM, magnification: 4 ×) and Masson (Scale bar: 100 µM, magnification: 10 ×) staining to assess pathological changes and collagen fiber deposition. **C** Immunofluorometric analysis was conducted to quantify CD31 expression in skin tissues. Scale bar: 100 µM, magnification: 10 ×. **D** Western blot assays were utilized to evaluate the protein expression levels of VEGFA, LC3 I/II, BECN1, p62, GSDMD-N, NLRP3, cleaved Caspase-1, and ASC in the skin tissues of DFU rats. Each experimental group consisted of eight rats (*n* = 8). *p < 0.05, **p < 0.01, ***p < 0.001

In comparision with to the non-treated group, SYD treatment led to a remarkable reduction in wound area and enhanced wound closure (Fig. 6A), even partially restoring the healing rate toward that observed in normal rats (Supplementary Figs.  3A and B). Histological examination revealed that SYD improved inflammatory infiltration and neovascularization (HE staining), as well as promoted collagen fiber deposition (Masson's trichrome staining) relative to controls (Fig. 6B). Moreover, immunofluorometric analysis demonstrated a marked upregulation of CD31 expression following SYD administration, indicative of enhanced angiogenesis (Fig. 6C). At the molecular level, SYD treatment led to increased expression of VEGFA, LC3 II/I, and BECN1, while concurrently decreasing the levels of p62, cleaved Caspase-1, GSDMD-N, NLRP3, and ASC in the skin tissues of DFU rats compared to controls (Fig. 6D). In addition, SYD-containing serum significantly increased METTL14 and BECN1 expression in fibroblasts under HG conditions, accompanied by enhanced autophagic activity and suppressed pyroptosis (Supplementary Fig. 4). Together, SYD accelerates DFU healing by promoting autophagy, suppressing pyroptosis, and stimulating angiogenic processes.

### METTL14 involvement in SYD-mediated regulation of autophagy, pyroptosis, and angiogenesis during DFU healing

**Fig. 7:**
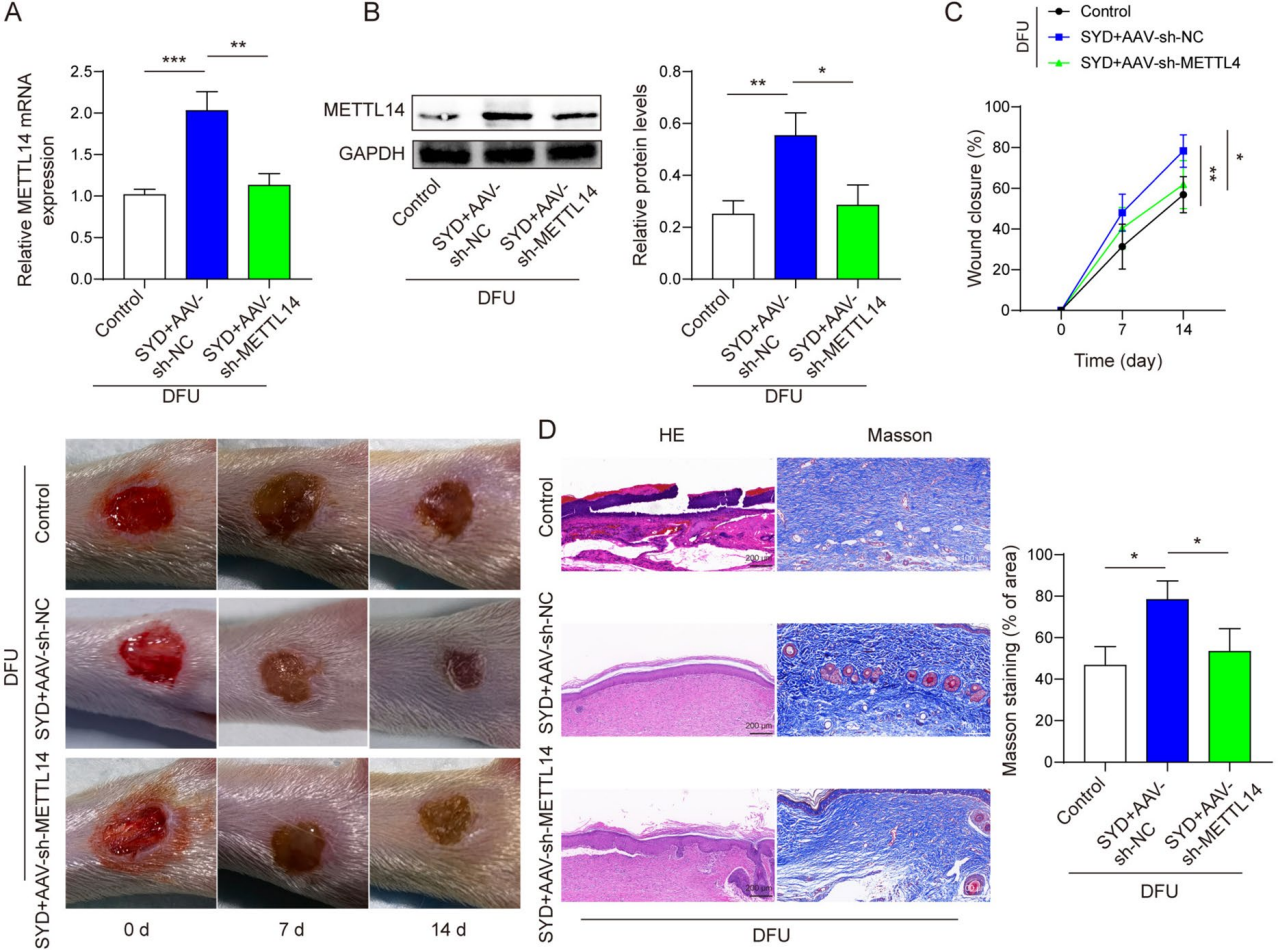
SYD facilitates the healing of DFU wounds through the modulation of METTL14. **A** qPCR analysis was conducted to assess the gene expression levels of METTL14 in skin tissues adjacent to the wound sites in DFU rat models. **B** Western blot analysis was performed to determine the protein expression levels of METTL14 in the same skin tissues. **C** Macroscopic evaluation of the wound area was carried out. **D** Histopathological examination (Scale bar: 200 µM, magnification: 4 ×) and Masson (Scale bar: 100 µM, magnification: 10 ×) staining of the skin tissues were conducted to assess tissue morphology and collagen deposition. Each experimental group consisted of eight rats (*n* = 8). *p < 0.05, **p < 0.01, ***p < 0.001

**Fig. 8:**
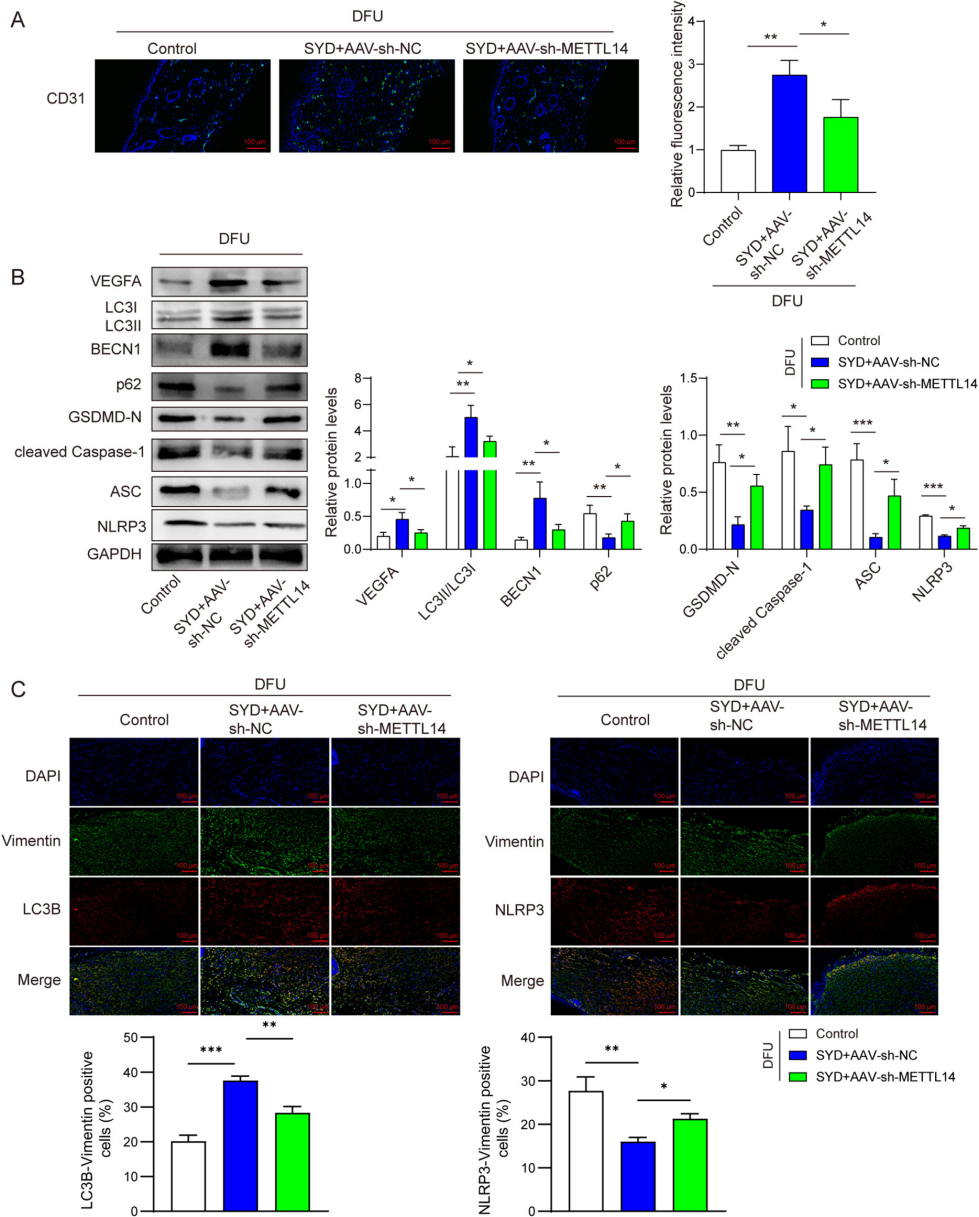
METTL14 is involved in the regulation of SYD concerning autophagy, pyroptosis, and angiogenesis in DFU rat models. **A** Immunofluorometric analysis of CD31 expression in skin tissue samples. Scale bar: 100 µM; magnification: 10 ×. **B** Western blot analysis was conducted to evaluate the expression levels of VEGFA, LC3 I/II, BECN1, p62, GSDMD-N, NLRP3, cleaved Caspase-1, and ASC in the skin tissues of DFU rats. **C** Representative immunofluorescence images showing LC3B and NLRP3 expression in fibroblasts of wound tissues. Nuclei were stained with DAPI (blue). Co-localization with Vimentin-positive cells indicates that these changes occur in fibroblasts. Scale bar: 100 μm. Magnification: 10 ×. Each experimental group consisted of eight rats (*n* = 8). *p < 0.05, **p < 0.01, ***p < 0.001

To elucidate the function of METTL14 in the protective effects of SYD on DFU rat models, shRNA AAV was employed to knock down METTL14 gene expression in these animals. The upregulation of METTL14 levels induced by SYD treatment in the skin tissues of DFU rats was significantly attenuated following METTL14 knockdown (Fig. 7A, B). Furthermore, METTL14 silencing markedly impaired the SYD-mediated enhancement of wound healing in DFU rats (Fig. 7C). Additionally, the knockdown partially reversed SYD-induced improvements in pathological changes of the skin and collagen fiber content (Fig. 7D). METTL14 suppression also inhibited the SYD-induced increase in CD31 expression (Fig. 8A). At the molecular level, METTL14 knockdown partially counteracted the SYD-driven upregulation of VEGFA, LC3 II/I, and BECN1, alongside the downregulation of cleaved Caspase-1, p62, GSDMD-N, NLRP3, and ASC in DFU rats (Fig. 8B). Immunofluorescence staining showed SYD increased LC3B expression and decreased NLRP3 expression in fibroblasts, while knockdown of METTL14 reverse these changes observed in METTL14 overexpression DFU rats (Fig. 8C). Collectively, these findings suggest that SYD facilitates wound healing in DFU rats by modulating autophagy, pyroptosis, and angiogenesis through the regulation of METTL14.

## Discussion

This investigation delineates the function of METTL14, a key m^6^A RNA methyltransferase, as an essential regulator of fibroblast role and wound healing in the context of DFUs. The findings reveal that METTL14 upregulates BECN1 expression via an m^6^A modification mechanism, thereby influencing the autophagy-pyroptosis pathway to mitigate excessive inflammatory cell death and promote tissue regeneration. Importantly, the study demonstrates that SYD enhances angiogenesis and accelerates wound repair by activating the METTL14/BECN1 signaling axis in a rat model of DFUs. Collectively, these results highlight the therapeutic promise of targeting METTL14 to improve clinical interventions for DFU treatment.

Chronic inflammation and impaired tissue repair are critical contributors to the pathogenesis of DFUs (Geng et al. 2021). Pyroptosis, mediated by inflammasomes, intensifies inflammatory responses and hinders the wound healing process (Geng et al. 2021; Mu et al. 2022; Wan et al. 2022).While, autophagy facilitates tissue regeneration by alleviating inflammatory responses and enhancing angiogenesis (Ji et al. 2023; Ren et al. 2022). Consequently, therapeutic strategies aimed at modulating the interplay between autophagy and pyroptosis hold significant potential for improving wound repairin diabetic patients (Ren et al. 2022). Emerging evidence suggests a functional crosstalk between autophagy and pyroptosis. Autophagy has been reported to negatively regulate inflammasome activation by promoting the degradation of inflammasome components, and by mitigating mitochondrial dysfunction. In this context, BECN1, as a key initiator of autophagy, may play a central role in suppressing pyroptosis. In this study, METTL14-mediated upregulation of BECN1 was related to enhanced autophagic activity and concurrent inhibition of the NLRP3/Caspase-1/GSDMD axis. Taken together, METTL14/BECN1-dependent autophagy may suppress pyroptosis through a coordinated regulatory mechanism, rather than representing parallel and independent processes. However, it is important to acknowledge the dualistic nature of autophagy in wound repair, wherein it can both support cell survival and induce apoptosis (Ripszky Totan et al. 2022). For instance, one study demonstrated that inhibition of AMPK/mTOR-mediated autophagy enhances wound healing (Wang et al. 2018), whereas another reported that treatment with bone marrow MSCs increased epidermal autophagy, thereby accelerating wound closure in diabetic wounds (Shi et al. 2022). In summary, autophagy appears to exert complex and sometimes contradictory effects on the wound healing process. The reason for this apparent discrepancy may be due to differences in cell types, such as fibroblasts versus endothelial progenitor cells, as well as variations in disease stages and microenvironmental conditions. Extensive evidence has established that epigenetic regulation are critically involved in the process of wound healing (Dubey et al. 2022; Geng et al. 2022). m^6^A modification, a prevalent RNA modification, influences various aspects of RNA metabolism, including cleavage, nuclear export, translation, and stability. Nevertheless, investigations into the association between m^6^A modification and wound repair in DFUs remain scarce. Liang et al. reported a significant reduction of the m^6^A reader protein YTHDC1 in the epidermis of both diabetic mice and patients (Liang et al. 2022). Futhermore,YTHDC1 was found to decrease the SQSTM1 mRNA stability while regulating SQSTM1-mediated autophagy in keratinocytes exposed to HG, thereby influencing wound healing progression in diabetes (Liang et al. 2022). Another study revealed that adipose-derived MSCs promote wound healing by promoting VEGFC-mediated angiogenesis via the METTL3/IGF2BP2-m^6^A axis in DFUs (Zhou et al. 2021). METTL14, a principal m^6^A methyltransferase, is downregulated under hyperglycemic conditions and has been implicated in diabetic nephropathy (Li et al. 2021; Xu et al. 2021). Its overexpression has been shown to inhibit pyroptosis and ameliorate diabetic cardiomyopathy (Meng et al. 2022). However, the functional role and underlying mechanisms of METTL14 in DFUs have yet to be elucidated. In our study, we observed a marked downregulation of METTL14 expression in fibroblasts derived from DFUs, concomitant with decreased global m^6^A levels and reduced BECN1 protein expression. Earlier findings suggest that METTL14 regulates m^6^A-methylated BECN1 mRNA, enhancing its translation by stabilizing the RNA in bone marrow MSCs undergoing osteogenic differentiation (He et al. 2022). In our study, METTL14 stabilizes BECN1 mRNA through m^6^A modification, thereby upregulating BECN1 protein expression. Importantly, mutation of a predicted m^6^A site in BECN1 significantly attenuated its ability to mediate the effects of METTL14 on autophagy and pyroptosis, providing functional evidence that this regulation is m^6^A-dependent rather than merely correlative. Furthermore, METTL14 overexpression improved autophagy, suppressed pyroptosis, and facilitated angiogenesis in vivo. In our animal model, the pro-healing effects induced by METTL14 overexpression were substantially abrogated following BECN1 knockdown, providing compelling in vivo evidence for a causal relationship whereby METTL14 exerts its effects via BECN1. Collectively, these findings suggest that SYD enhances autophagy, inhibits pyroptosis, promotes angiogenesis, and accelerates wound repair in DFUs by modulating METTL14-mediated m^6^A modification of BECN1.

Fibroblasts and endothelial cells are integral to the processes of angiogenesis and wound repair (Huang et al. 2020; Kim et al. 2021). Fibroblast-derived VEGFs activate endothelial cells, thereby facilitating their migration and promoting angiogenesis, which in turn accelerates the healing of DFUs (Chen et al. 2025; Rai et al. 2022). Earlier studies indicate that autophagy induction can attenuate pyroptosis mediated by inflammasome activation (Dai et al. 2017; Li et al. 2019). Therefore, the induction of autophagy to inhibit pyroptosis in fibroblasts may enhance endothelial cell angiogenesis and expedite wound healing. In line with this, our findings indicate that overexpression of METTL14 elevates autophagic activity and reduces pyroptosis in fibroblasts, thereby promoting endothelial cell motility and capillary-like structure formation. Collectively, these results suggest that METTL14 may stimulate VEGFA secretion from fibroblasts through the regulation of the autophagy-pyroptosis axis, leading to increased angiogenesis. Although VEGFA levels were significantly increased in conditioned medium, direct functional validation using VEGFA-neutralizing approaches is required to establish causality and will be addressed in future studies. This was further corroborated in an animal model, where METTL14 overexpression by AAV transfection significantly augmented the density of CD31-positive microvessels within wound tissues. It should be noted that AAV-mediated gene delivery is not strictly cell-type specific. Although our immunofluorescence data indicate that METTL14 is predominantly expressed in fibroblasts, potential effects in other cell types, including endothelial cells and immune cells, cannot be excluded. Future studies using cell type-specific genetic approaches are required to further clarify the contribution of METTL14 in different cellular compartments within the wound microenvironment. From a translational perspective, AAV-based gene therapy offers advantages such as sustained gene expression and relatively low immunogenicity. However, challenges including targeting specificity, delivery efficiency, and long-term safety remain to be addressed before clinical application in chronic wounds such as DFUs.

An additional noteworthy contribution of this research lies in elucidating a novel mechanistic basis for the therapeutic efficacy of the TCM formula SYD. Evidence from clinical investigations in traditional Chinese medicine supports the effectiveness of SYD in managing coronary heart disease, peripheral vascular conditions, and diabetes (Chen et al. 2021; Wang et al. 2015; Zhu et al. 2020). Prior investigations have demonstrated that SYD treatment enhances insulin sensitivity, reduces blood glucose levels, stabilizes atherosclerotic plaques, and subsequently improves cardiac function in diabetic murine models (Li et al. 2020; Peng et al. 2012). Furthermore, evidence suggests that SYD may facilitate the healing of DFUs through modulation of the Wnt/β-catenin signaling pathway (Zhao et al. 2017), a finding that aligns with our work. Our findings indicate that METTL14 expression in the wound skin tissues from DFU patients is upregulated following SYD treatment. Similarly, SYD administration increased METTL14 levels in the wound skin tissues of DFU rat models relative to controls. Notably, knockdown of METTL14 significantly abrogated the protective effects conferred by SYD in these DFU rats. Collectively, our findings suggest that SYD may promote autophagy activation, inhibit pyroptosis, enhance angiogenesis, and facilitate DFU healing through the regulation of METTL14-mediated m^6^A modification of BECN1. However, the specific bioactive component(s) and upstream regulatory mechanisms remain to be elucidated.

Given the multi-component nature of SYD, its therapeutic effects are unlikely to be attributed to a single molecular pathway. Previous studies have demonstrated that SYD and its bioactive compounds possess anti-inflammatory and antioxidative activities, playing an critical role in improving the impaired wound microenvironment in diabetes. In addition, modulation of metabolic homeostasis may further contribute to tissue repair. In this context, our findings identify the METTL14/BECN1 axis as one important mechanism linking epigenetic regulation to the autophagy–pyroptosis balance in fibroblasts. However, it is likely that this pathway operates in concert with other biological processes regulated by SYD. Future studies are warranted to dissect the relative contribution of individual components and signaling pathways underlying the therapeutic effects of SYD.

## Conclusions

**Fig. 9:**
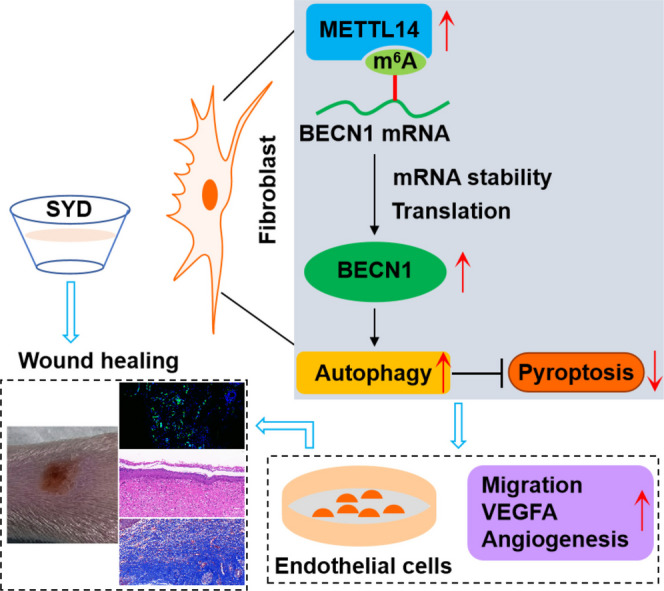
A schematic representation elucidating the biological function of the METTL14/BECN1 axis and SYD in regulating autophagy, pyroptosis, and angiogenesis throughout the process of DFU wound healing

In summary (Fig. 9), this study underscores the pivotal function of METTL14 in modulating the autophagy-pyroptosis axis within fibroblasts, thereby facilitating wound repair in DFUs. Specifically, METTL14 enhances BECN1 expression through m^6^A modification of its mRNA, thereby modulating autophagy and pyroptosis. Although SYD was observed to enhance DFU healing, its therapeutic effects appear to be associated with increased METTL14 expression. These results indicate that both METTL14 and BECN1 represent promising therapeutic targets for DFUs, and further investigation into the role of METTL14 in DFU pathophysiology may provide assistance for the development of innovative therapy approaches.

## Supplementary Information

Below is the link to the electronic supplementary material.

Supplementary file1 (DOCX 3828 KB)

## Data Availability

The datasets used or analyzed during the current study are available from the corresponding author on reasonable request.
