# Supplementary material for: METTL14 modulates the autophagy-pyroptosis pathway in fibroblasts by modifying BECN1 through m6A methylation to promote wound healing in DFUs and SYD treatment
Source: Cell Biol Toxicol. 2026 May 1;42(1):75. doi: 10.1007/s10565-026-10196-x (PMC13249717; doi:10.1007/s10565-026-10196-x)
Supplement: Supplementary file 1 — Supplementary file1 (DOCX 3828 KB) [file 10565_2026_10196_MOESM1_ESM.docx]

**Supplementary Figures and Legends**


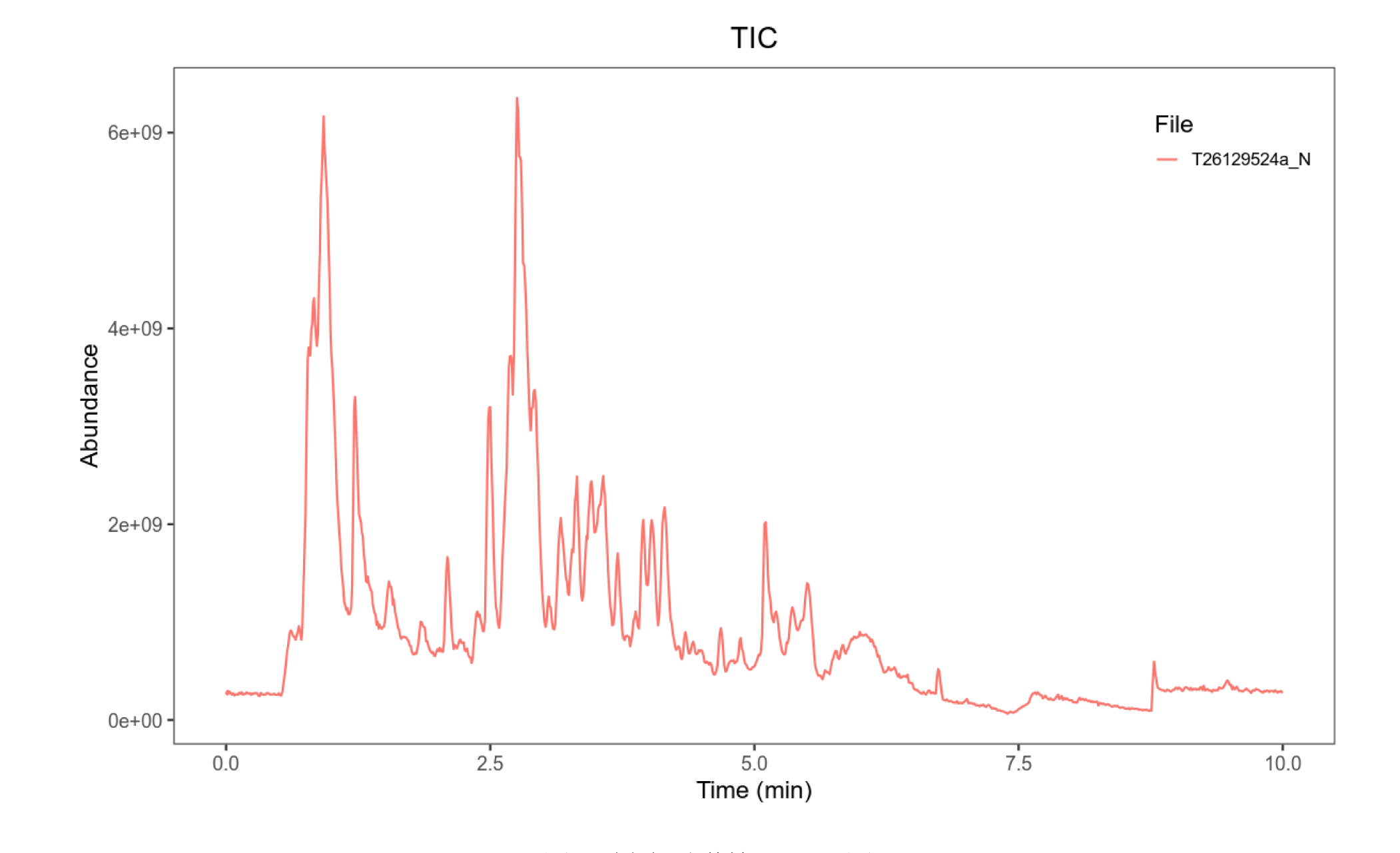


**Supplementary Figure 1. UPLC-MS/MS analysis identified chemical constituents in SYD.**

**
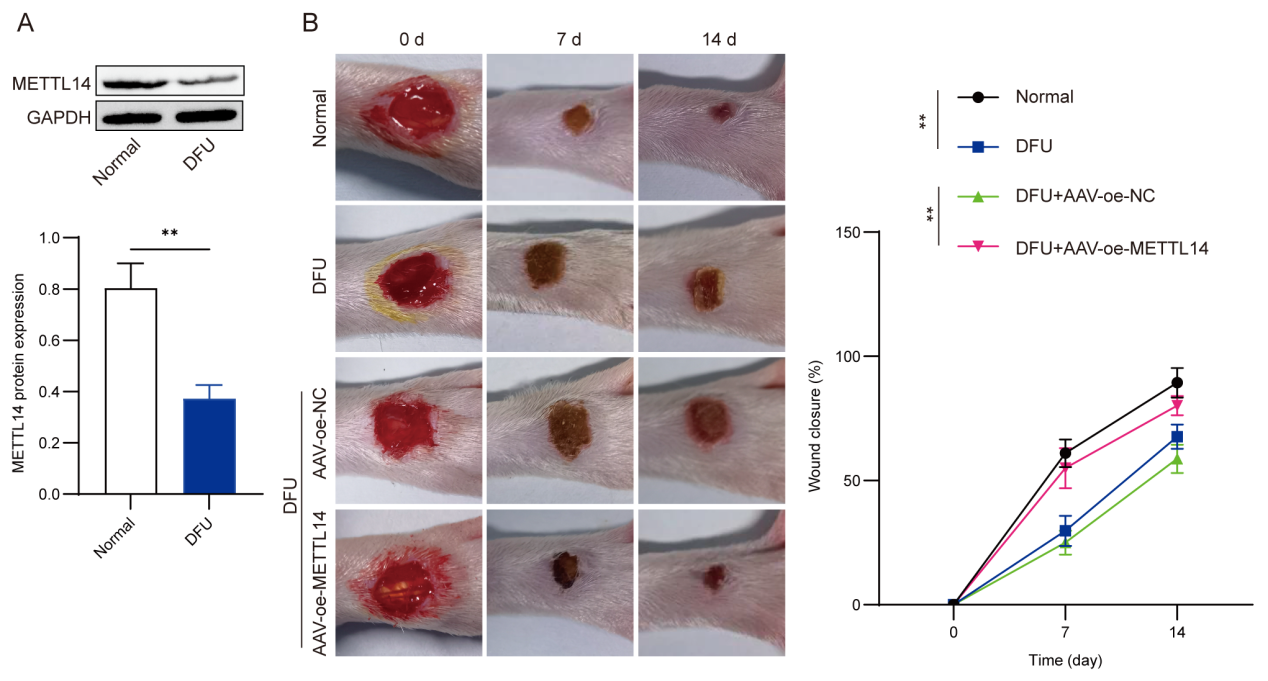
**

**Supplementary Figure 2. Overexpression of METTL14 promotes wound healing in DFU rats.** (A) Western blot analysis was conducted to evaluate METTL14 protein levels in the skin tissues. (B) The wound areas of DFU rats were monitored and documented. Each experimental group consisted of eight rats (n = 8). **p <0.01.


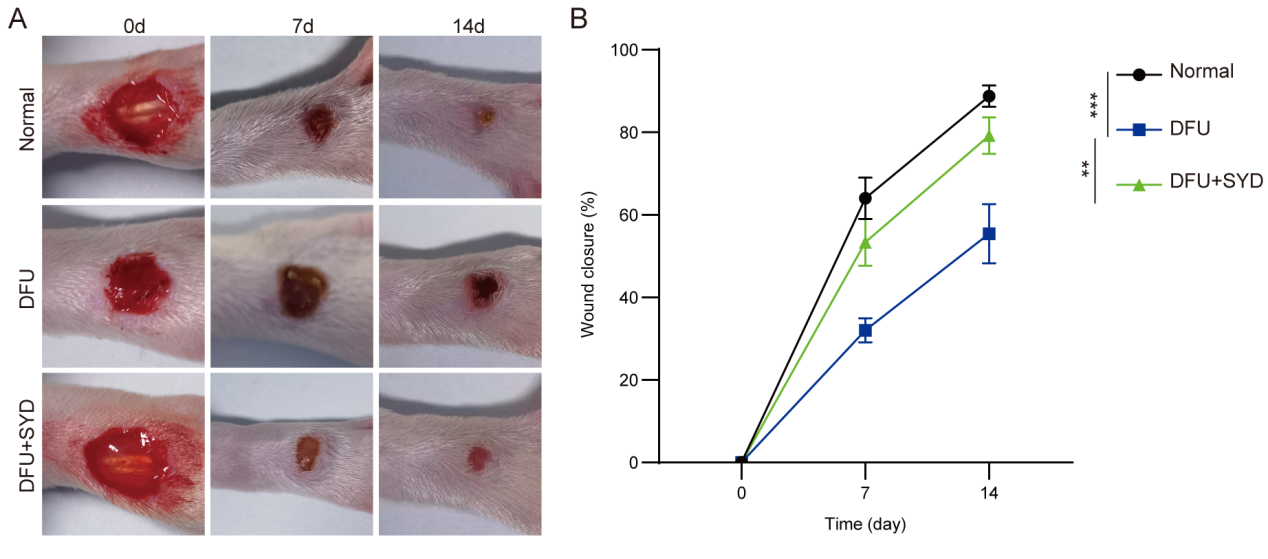


**Supplementary Figure 3. SYD facilitates wound healing in DFU rats.** (A) The wound areas of DFU rats were imaged. (B) The wound closure of DFU rats were calculated. Each experimental group consisted of eight rats (n = 8). **p <0.01, ***p <0.001.


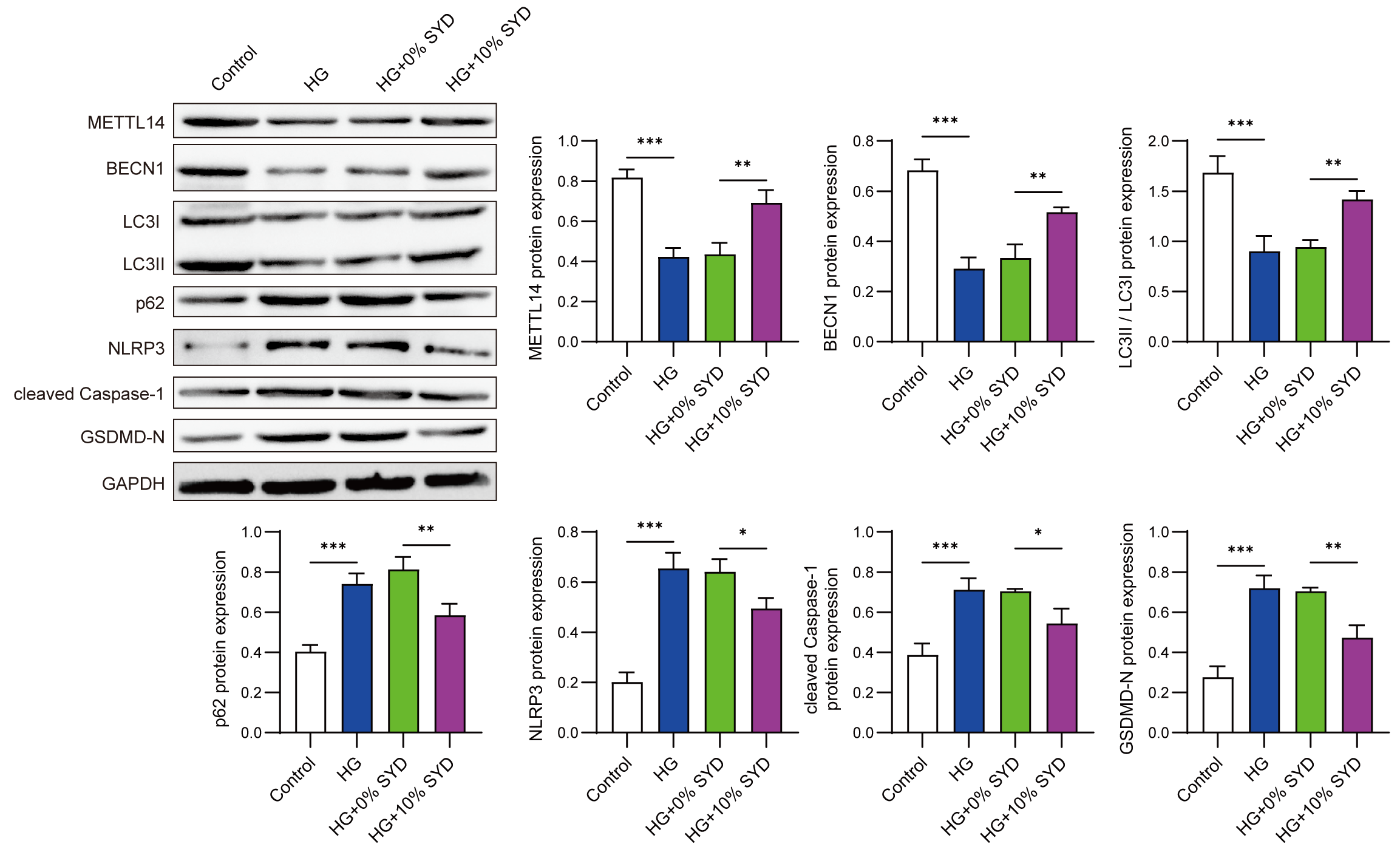


**Supplementary Figure 4. Effects of SYD-containing serum on the expression of METTL14 and autophagy-pyroptosis pathway.** Fibroblasts were treated with HG and SYD-containing serum for 48 hours. Western blot analysis was performed to measure the expression levels of METTL14, LC3 II/I, BECN1, p62, GSDMD-N, NLRP3, and cleaved Caspase-1. n = 3. *p <0.05, **p <0.01, ***p <0.001.


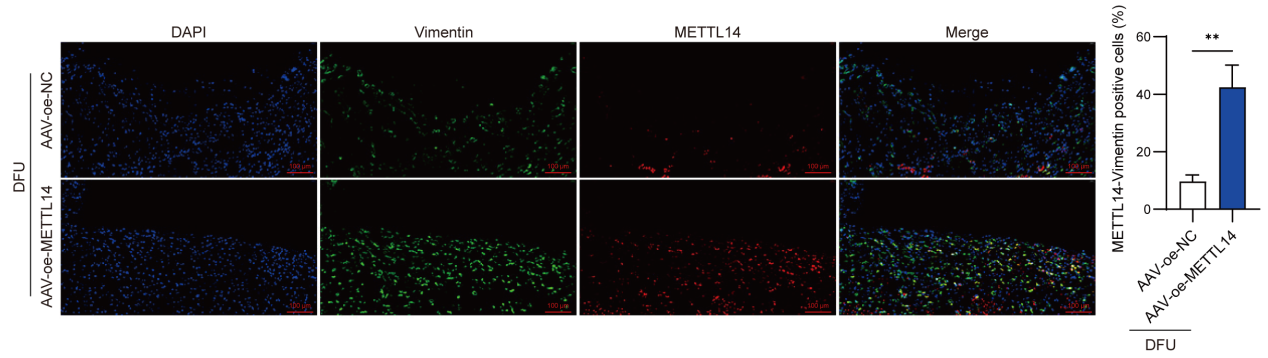


**Supplementary Figure 5. Expression of METTL14 in wound fibroblasts of DFU rats.** Fibroblasts were treated with HG and SYD-containing serum for 48 hours.Representative immunofluorescence images showing METTL14 expression in in fibroblasts of wound tissues. Nuclei were stained with DAPI (blue). Co-localization with Vimentin-positive cells indicates that these changes occur in fibroblasts. Scale bar: 100 μm. Magnification: ×100. Each experimental group consisted of eight rats (n = 8). **p <0.01.


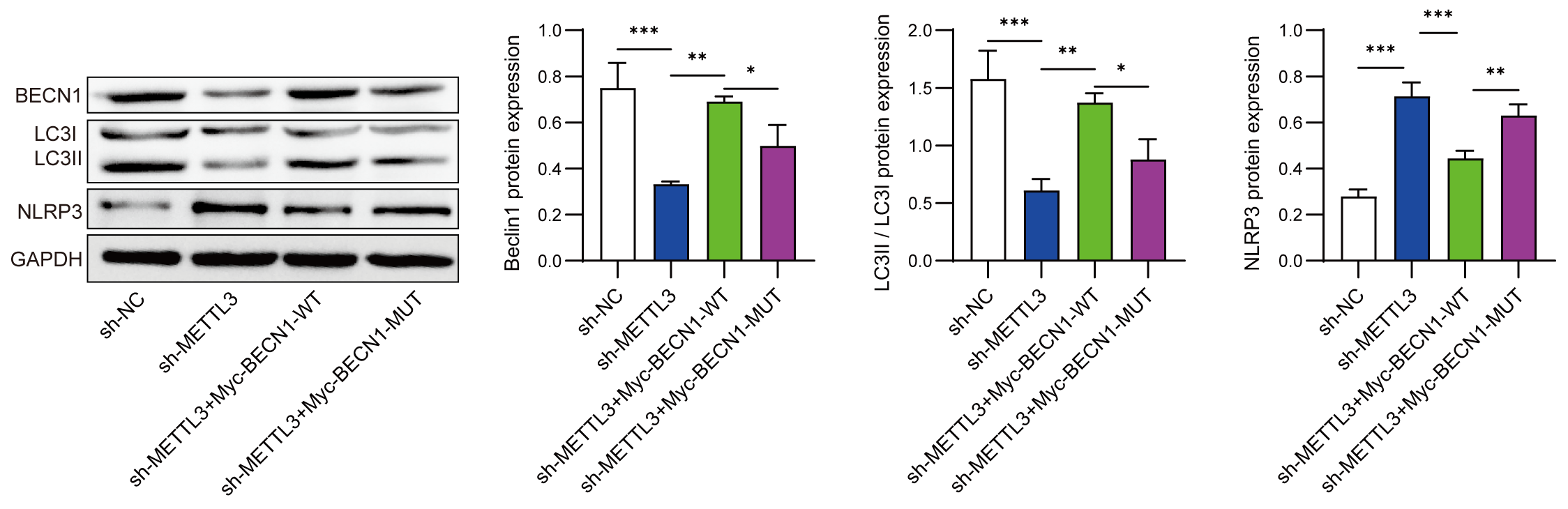


**Supplementary Figure 6. The impact of BECN1 site mutation on the regulatory role of METTL14 in the processes of autophagy and pyroptosis.** Western blot analysis was performed to measure the expression levels of BECN1, LC3 II/I, and NLRP3 in fibroblasts. n = 3. *p <0.05, **p <0.01, ***p <0.001.
